# Body Composition Remodelling During GLP‐1‐Based Therapy: A Systematic Review and Meta‐Analysis Using a Hierarchical Physiological Framework

**DOI:** 10.1111/dom.71220

**Published:** 2026-08-17

**Authors:** Maria Luizza Lopes Menezes, Maria Eduarda Güntzel Chiappa, Rodrigo Fuentes Kloss, Marcelo Tuesta, Paula Santos, Alberto Souza Sá Filho, Gaspar R. Chiappa

**Affiliations:** ^1^ Secretariat of Health of the Federal District Brasília Brazil; ^2^ School of Public Health of the Federal District Brasília Brazil; ^3^ Federal University of Rio Grande do Sul Porto Alegre Brazil; ^4^ Health Sciences School, Faculty of Medicine Pontificia Universidad Católica de Chile Santiago Chile; ^5^ Exercise and Rehabilitation Sciences Institute, School of Physical Therapy Faculty of Rehabilitation Sciences, Universidad Andres Bello Santiago Chile; ^6^ Graduate Program in Human Movement and Rehabilitation of Evangelical University of Goiás Goiás Brazil; ^7^ Faculty of Health Sciences Universidad Autónoma de Chile Santiago Chile

**Keywords:** body composition, GLP‐1, meta‐analysis, systematic review, type 2 diabetes, weight control

## Abstract

**Background:**

Body composition changes during glucagon‐like peptide‐1 (GLP‐1)‐based therapy remain incompletely characterized, particularly regarding their effects on adiposity, muscle quantity, and muscle quality. We conducted a systematic review and meta‐analysis using a predefined physiological framework to evaluate body composition remodelling during GLP‐1‐based therapy.

**Methods:**

We searched PubMed, Embase, and Cochrane CENTRAL from inception through May 2026 for studies evaluating GLP‐1 receptor agonists or dual incretin therapies reporting longitudinal body composition outcomes in adults. Outcomes were classified into adiposity, muscle quantity, muscle quality, and body weight domains. Primary analyses prioritized comparative evidence and included one physiologically coherent outcome per study and domain. Random‐effects meta‐analyses were performed using restricted maximum likelihood estimation with Hartung‐Knapp adjustment.

**Results:**

Nineteen studies met the eligibility criteria, including comparative and exploratory single‐arm investigations. Three comparative studies contributed to the primary adiposity meta‐analysis and two to the primary muscle quantity meta‐analysis. Comparative analyses suggested a direction toward greater reductions in adiposity during GLP‐1‐based therapy. However, only three studies contributed to this analysis, and the pooled estimate was characterized by substantial between‐study heterogeneity and a wide confidence interval crossing the null. Evidence regarding muscle quantity was based on two comparative studies and remained highly heterogeneous and imprecise. Muscle quality outcomes could not be quantitatively synthesized because of methodological heterogeneity and incomplete variance reporting and were therefore evaluated narratively. Exploratory single‐arm analyses showed reductions in adiposity and body weight, whereas changes in muscle quantity varied across studies.

**Conclusions:**

Current comparative evidence suggests a possible direction toward greater reductions in adiposity during GLP‐1‐based therapy. However, the limited number of comparative studies, substantial between‐study heterogeneity, wide confidence intervals, and low to very low certainty of evidence preclude definitive conclusions regarding preferential fat loss or preservation of skeletal muscle health. Reductions in lean mass should not be interpreted as synonymous with sarcopenia, but the available evidence is also insufficient to exclude clinically relevant muscle loss in susceptible populations. These findings should therefore be considered hypothesis‐generating and require confirmation in adequately powered comparative studies incorporating standardized assessments of muscle quantity, muscle quality, strength, and physical performance.

**PROSPERO Registration:**

CRD420261411693.

## 1 Introduction

Glucagon‐like peptide‐1 (GLP‐1) receptor agonists and dual incretin therapies have transformed the management of obesity and Type 2 diabetes by producing substantial and sustained weight loss while improving glycemic control and cardiometabolic risk factors. Large randomized trials have consistently demonstrated clinically meaningful reductions in body weight with semaglutide and tirzepatide, accompanied by improvements in cardiovascular and metabolic outcomes [1, 2, 3]. As the use of incretin‐based therapies expands, increasing attention has focused on the composition of weight loss and its implications for musculoskeletal health.

Weight reduction is invariably accompanied by changes in lean tissue; however, the physiological significance of these changes remains controversial. Conventional measures of lean mass derived from dual‐energy x‐ray absorptiometry (DXA) do not directly quantify skeletal muscle tissue and encompass non‐contractile components such as body water, connective tissue, and organ mass [4]. Consequently, reductions in lean mass cannot be automatically interpreted as skeletal muscle loss or treatment‐induced sarcopenia. Furthermore, obesity itself is associated with impaired muscle quality and ectopic fat accumulation, suggesting that improvements in muscle composition may occur despite decreases in absolute lean tissue [5, 6, 7]. Existing studies have used heterogeneous imaging modalities and outcome definitions, often combining adiposity and muscle‐related measures without distinguishing between muscle quantity and muscle quality, thereby complicating interpretation of body composition changes during incretin‐based therapy.

To date, no systematic review has comprehensively evaluated body composition remodelling during GLP‐1‐based therapy using a predefined physiological framework that distinguishes adiposity, muscle quantity, and muscle quality while prioritizing comparative evidence. We therefore conducted a systematic review and meta‐analysis to characterize changes across these domains during treatment with GLP‐1 receptor agonists and dual incretin therapies. By applying an analytical hierarchy and restricting primary analyses to one physiologically coherent outcome per study and domain, we sought to provide a more rigorous assessment of tissue remodelling and its implications for skeletal muscle health.

## 2 Methods

### 2.1 Study Design and Registration

We conducted a systematic review and meta‐analysis in accordance with the Preferred Reporting Items for Systematic Reviews and Meta‐Analyses (PRISMA) 2020 statement [8]. We developed the review protocol a priori and prospectively registered it in the International Prospective Register of Systematic Reviews (PROSPERO; CRD420261411693). We structured the analyses to characterize body composition remodelling during GLP‐1‐based therapy using a predefined physiological framework.

### 2.2 Search Strategy and Study Selection

We systematically searched PubMed, Embase, and Cochrane CENTRAL from database inception through May 2026. The complete search strategies for all databases are provided in Table S2. We used combinations of controlled vocabulary and free‐text terms related to glucagon‐like peptide‐1 receptor agonists, dual incretin therapies, semaglutide, tirzepatide, body composition, adiposity, lean mass, skeletal muscle, and sarcopenia.

Two reviewers independently screened titles and abstracts and subsequently assessed full texts for eligibility. Disagreements were resolved by consensus.

We included randomized controlled trials and observational studies enrolling adults receiving GLP‐1 receptor agonists or dual incretin therapies and reporting longitudinal body composition outcomes. We excluded studies involving pediatric populations, conference abstracts, reviews, case reports, animal studies, and studies lacking sufficient quantitative data.

### 2.3 Outcome Classification Framework

We classified outcomes according to a predefined physiological hierarchy comprising adiposity, muscle quantity, muscle quality, and body weight. Adiposity outcomes included total fat mass, percentage body fat, visceral adipose tissue, and related measures. Muscle quantity outcomes included appendicular lean mass, total lean mass, fat‐free mass, and skeletal muscle mass. Muscle quantity outcomes included appendicular lean mass, total lean mass, fat‐free mass, and skeletal muscle mass. These measures were considered proxy measures of skeletal muscle quantity and were not interpreted as direct assessments of skeletal muscle health, muscle quality, muscle strength, physical performance, or sarcopenia. Body weight outcomes were considered separately because they represent whole‐body changes rather than specific tissue compartments.

To preserve physiological coherence and statistical independence, we selected one outcome per study and domain for the primary comparative analyses. When multiple measurements were available, we prioritized outcomes according to the predefined analytical hierarchy described in Table S5. A study‐level summary of the selected outcome, measurement unit, analytical role, variance source, and rationale for outcome selection is provided in Table S7.

### 2.4 Data Extraction

Two investigators independently extracted study characteristics, participant demographics, intervention details, duration of follow‐up, imaging modality, and outcome data. We recorded means, standard deviations, and sample sizes whenever available. For studies reporting medians or alternative summary statistics, we applied established conversion methods when appropriate [9].

### 2.5 Risk of Bias Assessment

We assessed randomized controlled trials using the Cochrane Risk of Bias 2 tool [10] and observational studies using the Risk Of Bias In Non‐randomized Studies of Interventions (ROBINS‐I) tool [11]. Summary assessments are presented in Figures S1 and S2, and detailed assessments are provided in Table S3.

### 2.6 Statistical Analysis

We prioritized comparative studies for the primary analyses. Given the limited number of available comparative studies, these analyses were considered exploratory, whereas single‐arm studies were synthesized separately as exploratory evidence. We performed random‐effects meta‐analyses using restricted maximum likelihood (REML) estimation with Hartung‐Knapp adjustment to account for uncertainty in between‐study variance [12, 13].

Because body composition outcomes were measured using different scales and imaging modalities, we calculated pooled standardized mean differences (Hedges' *g*) with corresponding 95% confidence intervals. We quantified statistical heterogeneity using Cochran's *Q* statistic and the *I* ^2^ statistic. We interpreted *I* ^2^ values qualitatively according to conventional thresholds.

Given the limited number of comparative studies, we restricted primary quantitative analyses to adiposity and muscle quantity domains. Evidence regarding muscle quality was synthesized narratively because available studies used heterogeneous methodologies and reported insufficient data for quantitative pooling.

We conducted exploratory single‐arm meta‐analyses for adiposity, muscle quantity, and body weight outcomes. To explore the influence of individual studies and potential sources of heterogeneity, we performed leave‐one‐out sensitivity analyses and Baujat plots for the primary comparative adiposity analysis (Figures S7 and S8), with individual omission estimates summarized in Table S6. Given the limited number of comparative studies, these analyses were considered supportive diagnostic procedures and were interpreted descriptively rather than as formal evidence of robustness.

### 2.7 Certainty of Evidence

We assessed the certainty of evidence for the primary comparative outcomes using the GRADE framework [14]. Certainty was rated as low for the primary comparative adiposity outcome and very low for the primary comparative lean tissue/muscle quantity proxy outcome. Adiposity evidence was downgraded for serious inconsistency and serious imprecision. Muscle quantity proxy evidence was downgraded for serious inconsistency, serious indirectness, and serious imprecision because only two studies were available and the outcomes consisted of lean tissue–based proxy measures rather than direct assessments of skeletal muscle health. Risk of bias was not rated as serious for either outcome because the contributing evidence originated from randomized comparative trials with overall RoB 2 judgements of some concerns. Detailed effect estimates, participant numbers, heterogeneity statistics, and domain‐specific justifications are provided in Table S4. Publication bias could not be formally assessed because of the small number of studies included in each meta‐analysis.

### 2.8 Software

We performed all statistical analyses using R version 4.5.0 (R Foundation for Statistical Computing, Vienna, Austria). We conducted meta‐analyses using the meta and metafor packages [15, 16].

## 3 Results

### 3.1 Study Selection

The literature search identified 3427 records, including 1242 from PubMed/MEDLINE, 1986 from Embase, and 199 from Cochrane CENTRAL. After removal of 1187 duplicates, 2240 records underwent title and abstract screening. We excluded 2097 records during screening, and 143 reports were sought for retrieval. Twelve reports could not be retrieved, leaving 131 full‐text reports assessed for eligibility. Of these, 112 reports were excluded because of wrong population, wrong intervention, wrong outcomes, wrong study design, unavailable usable data, or duplicate publication. Ultimately, 19 studies met the eligibility criteria and were included in the systematic review (Figure 1) [6, 7, 17, 18, 19, 20, 21, 22, 23, 24, 25, 26, 27, 28, 29, 30, 31, 32, 33, 34].

**FIGURE 1:**
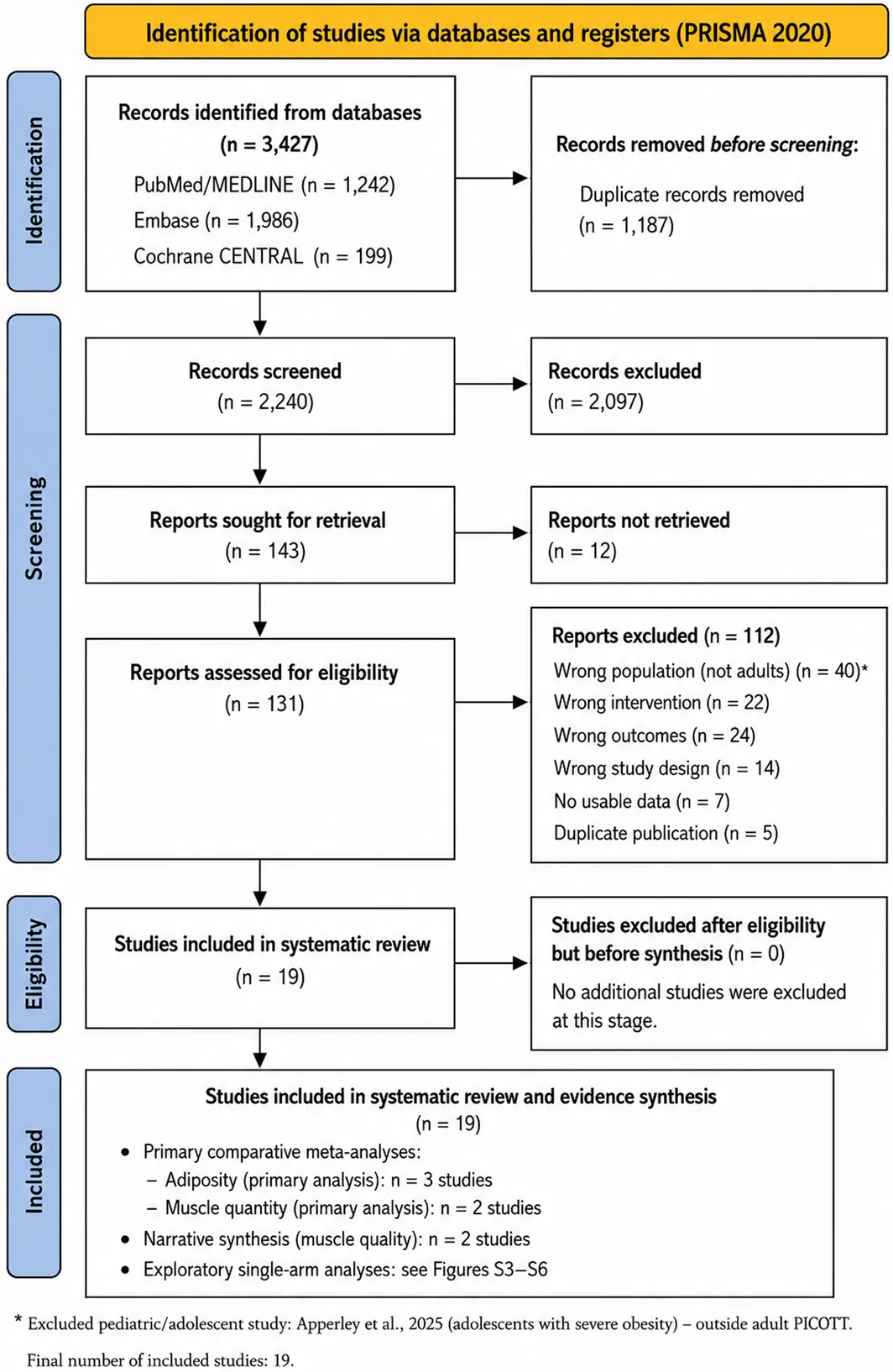
PRISMA 2020 flow diagram of the study selection process.

### 3.2 Study Characteristics

The 19 included studies comprised randomized controlled trials, comparative observational studies, and exploratory single‐arm investigations evaluating GLP‐1 receptor agonists or dual incretin therapies in adults with obesity and/or Type 2 diabetes. Body composition was assessed predominantly using dual‐energy x‐ray absorptiometry (DXA), although magnetic resonance imaging (MRI), computed tomography (CT), and bioelectrical impedance analysis (BIA) were also used. Table 1 summarizes the principal study characteristics, whereas expanded study characteristics are provided in Table S1. The predefined outcome classification framework and analytical hierarchy are presented in Table S5. Study‐level implementation of this hierarchy, including the selected outcome, analytical role, and variance source for each study, is summarized in Table S7.

**TABLE 1:** Study characteristics and analytical hierarchy.

Study	Design	Population	*n*	Drug class	Intervention	Comparator	Follow‐up	Assessment method	Outcome domain(s)	Extracted outcomes	Analytical role
Coskun et al. [17]	Phase 2 randomized DXA substudy	T2D	Up to 126 retatrutide; 29 placebo; 34 dulaglutide	GLP‐1/GIP/glucagon triagonist	Retatrutide pooled doses	Placebo or dulaglutide	36 weeks	DXA	Adiposity	Total fat mass	Primary comparative
Look et al. [18]	SURMOUNT‐1 randomized DXA substudy	Obesity or overweight without diabetes	160	GLP‐1/GIP dual agonist	Tirzepatide pooled doses	Placebo	72 weeks	DXA	Weight; adiposity; muscle quantity	Body weight; fat mass; lean mass	Primary comparative
McCrimmon et al. [19]	Randomized DXA substudy	T2D receiving metformin	114	GLP‐1RA	Semaglutide 1.0 mg	Canagliflozin 300 mg	52 weeks	DXA	Adiposity; muscle quantity	Total fat mass; total lean mass; visceral fat mass; total fat mass percentage; total lean mass percentage	Primary comparative
Pandey et al. [6]	Prespecified secondary analysis of randomized trial	Adults with overweight or obesity without diabetes	128	GLP‐1RA	Liraglutide 3.0 mg plus lifestyle intervention	Placebo plus lifestyle intervention	Median 36 weeks	MRI	Muscle quality; muscle quantity	Thigh muscle fat; muscle volume z score	Narrative muscle‐quality evidence
Feng et al. [20]	Randomized open‐label active‐comparator trial	T2D plus NAFLD	85	GLP‐1RA	Liraglutide 1.8 mg	Metformin or gliclazide	24 weeks	DXA	Adiposity; muscle quantity	Total fat mass; total lean tissue; trunk fat; limb fat; android fat	Supportive comparative
Heise et al. [7]	Phase 1 placebo‐controlled trial	T2D	76	GLP‐1/GIP dual agonist	Tirzepatide 15 mg	Placebo	28 weeks	MRI‐based body composition assessment	Adiposity	Fat mass	Supportive comparative
Sattar et al. [21]	Post hoc MRI substudy of randomized trial	T2D plus overweight or obesity	246	GLP‐1/GIP dual agonist	Tirzepatide pooled doses	Insulin degludec	52 weeks	MRI	Muscle quality; muscle quantity	Intramuscular fat; muscle volume; muscle volume z score	Supportive comparative
Yang et al. [22]	Retrospective comparative cohort	T2D plus CKD	73	GLP‐1RA plus SGLT2 inhibitor	SGLT2 inhibitor plus semaglutide	SGLT2 inhibitor alone	≥ 6 months	BIA	Adiposity; muscle quantity	FMI; muscle mass; SMI	Supportive observational comparative
Basolo et al. [23]	Prospective single‐arm study	Adults with obesity	11	GLP‐1RA	Liraglutide	None	6 months	DXA	Weight; adiposity; muscle quantity	Body weight; fat mass; lean tissue	Exploratory single‐arm
Bhatti et al. [24]	Retrospective real‐world cohort	Overweight or obesity	269	GLP‐1RA; GLP‐1/GIP dual agonist	Semaglutide or tirzepatide plus lifestyle program	None	6 and 12 months	BIA/InBody 370S	Weight; adiposity; muscle quantity	Weight; BMI; fat mass; skeletal muscle mass	Exploratory single‐arm
Chun et al. [25]	Retrospective real‐world cohort	Commercial weight‐management setting	94	GLP‐1RA	Compounded semaglutide/cyanocobalamin	None	3 months	BIA/InBody 570	Weight; adiposity; muscle quantity	Weight; fat mass; lean body mass; skeletal muscle mass	Exploratory single‐arm
Gjersdal et al. [26]	Single‐arm case series	Hypothalamic obesity after craniopharyngioma	4	GLP‐1RA	Semaglutide	None	6–24 months	DXA	Weight; adiposity; muscle quantity	Weight; BMI; fat mass; lean mass	Exploratory single‐arm
Ishikawa et al. [27]	Single‐arm add‐on cohort	T2D‐associated NAFLD/NASH	15	GLP‐1RA plus SGLT2 inhibitor	Oral semaglutide plus luseogliflozin	None	48 weeks	CT	Weight; adiposity	BMI; SATI; VATI	Exploratory single‐arm
Nelson et al. [33]	Retrospective intrapatient CT cohort	Adults using semaglutide	241	GLP‐1RA	Semaglutide	None	Variable	Automated CT	Weight; adiposity; muscle quantity; muscle quality	VAT; SAT; skeletal muscle area; attenuation; IMAT	Exploratory single‐arm
Rodriguez Jimenez et al. [29]	Retrospective quasi‐experimental study	T2D plus obesity	88	GLP‐1RA	Subcutaneous or oral semaglutide	None	24 weeks	BIA	Weight; adiposity; muscle quantity	Fat mass; lean mass; skeletal muscle mass	Exploratory single‐arm
Uchiyama et al. [30]	Retrospective longitudinal study	Japanese adults with T2D	25	GLP‐1RA	Oral semaglutide	None	12 and 24 weeks	BIA	Weight; adiposity; muscle quantity	Whole‐body fat; lean mass; SMI	Exploratory single‐arm
Volpe et al. [31]	Prospective real‐life study	T2D	40	GLP‐1RA	Once‐weekly subcutaneous semaglutide	None	26 weeks	SMF‐BIA/ultrasound/handgrip	Weight; adiposity; muscle quantity; muscle quality	VAT; FMI; SMI; Phase angle (cellular integrity surrogate); handgrip strength (functional context); handgrip strength	Exploratory single‐arm
Volpe et al. [31]	Prospective real‐life study	T2D	32	GLP‐1RA	Oral semaglutide	None	26 weeks	SMF‐BIA	Adiposity; muscle quantity; muscle quality	Fat mass; fat‐free mass; SMI; phase angle	Exploratory single‐arm
Xiang et al. [34]	Retrospective real‐world cohort	Chinese adults with obesity without diabetes	43	GLP‐1RA	Semaglutide plus lifestyle intervention	None	24 weeks	BIA/InBody S10	Weight; adiposity; muscle quantity	Body weight; fat mass; visceral fat area; skeletal muscle mass; SMI	Exploratory single‐arm

*Note:* The *n* column refers to the body‐composition analysis sample when reported and may differ from the parent trial sample size. Primary comparative analyses selected one outcome per study and domain according to the prespecified hierarchy to avoid double counting. Supportive comparative studies were retained to provide contextual evidence but were not included in the primary comparative meta‐analyses unless prespecified according to the analytical hierarchy. Handgrip strength in Volpe et al. [32] was extracted as exploratory functional context and was not treated as a separate pooled domain. Muscle quality outcomes were synthesized narratively because the available studies differed substantially in imaging modality, anatomical site, outcome definition, measurement scale, and variance reporting, precluding meaningful quantitative pooling.

Abbreviations: BIA, bioelectrical impedance analysis; CKD, chronic kidney disease; CT, computed tomography; DXA, dual‐energy x‐ray absorptiometry; FMI, fat mass index; GIP, glucose‐dependent insulinotropic polypeptide; GLP‐1, glucagon‐like peptide‐1; GLP‐1RA, glucagon‐like peptide‐1 receptor agonist; IMAT, intermuscular adipose tissue; MRI, magnetic resonance imaging; NAFLD, nonalcoholic fatty liver disease; NASH, nonalcoholic steatohepatitis; SAT, subcutaneous adipose tissue; SATI, subcutaneous adipose tissue index; SGLT2, sodium‐glucose cotransporter 2; SMF‐BIA, segmental multifrequency bioelectrical impedance analysis; SMI, skeletal muscle index; T2D, Type 2 diabetes; VAT, visceral adipose tissue; VATI, visceral adipose tissue index.

### 3.3 Risk of Bias

Among randomized studies, most domains were judged as low risk or raised some concerns according to the Cochrane Risk of Bias 2 tool (Figure S1). Observational comparative and single‐arm studies showed greater susceptibility to confounding and selection bias, leading predominantly to serious overall risk assessments using ROBINS‐I (Figure S2). Detailed assessments are provided in Table S3.

### 3.4 Primary Comparative Analyses

#### 3.4.1 Adiposity

Three studies contributed to the primary comparative analysis of adiposity (Figure 2). Individual studies consistently favoured greater reductions in adiposity during GLP‐1‐based therapy; however, the pooled estimate was characterized by substantial between‐study heterogeneity and wide confidence intervals, limiting the precision of the overall effect estimate. Leave‐one‐out sensitivity analyses demonstrated variability in pooled estimates after sequential omission of individual studies, reflecting the limited number of available comparative studies (Figure S7 and Table S6). The Baujat plot identified McCrimmon et al., 2020 as the principal contributor to heterogeneity and study influence (Figure S8).

**FIGURE 2:**
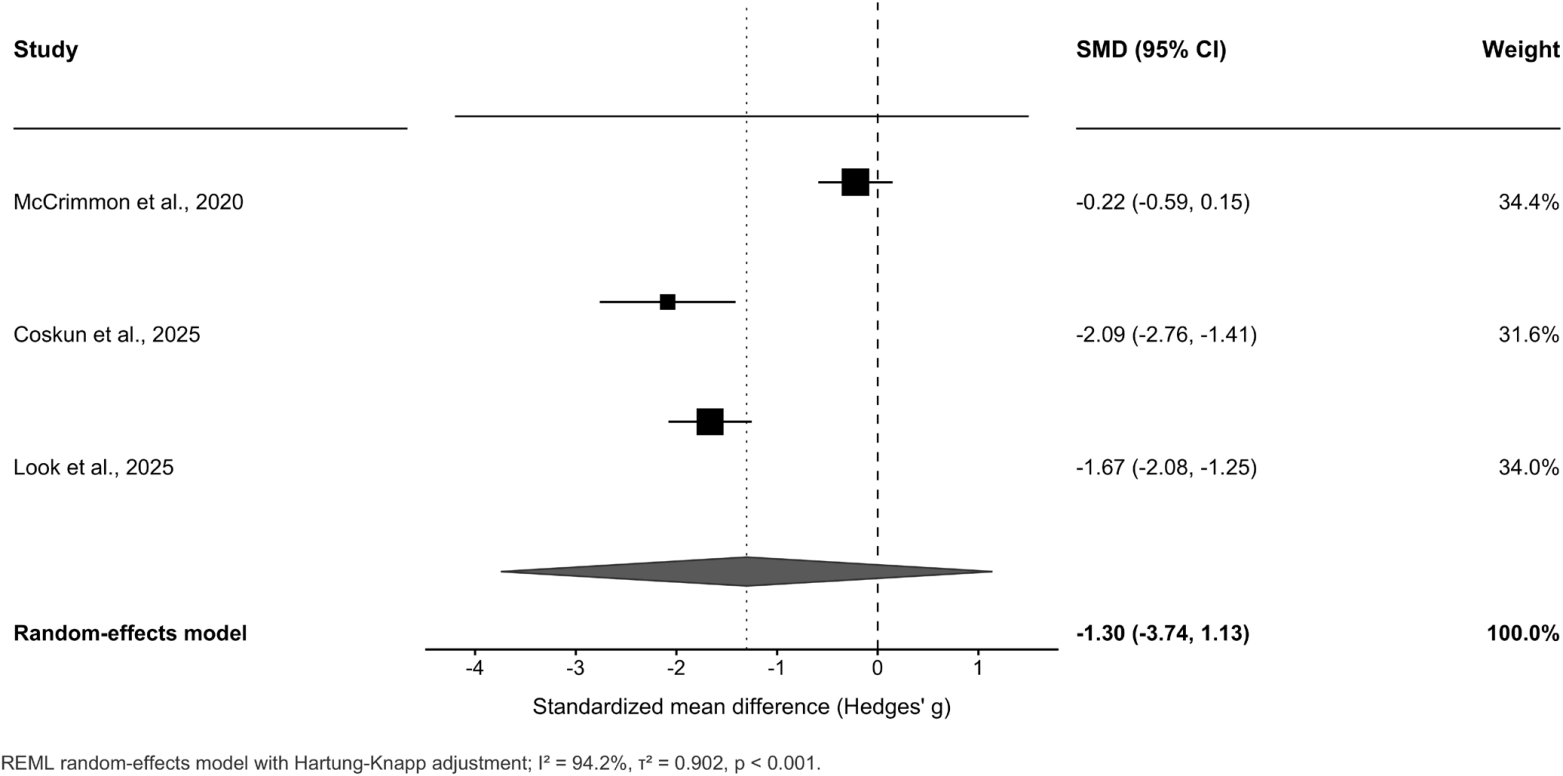
Forest plot of the primary comparative meta‐analysis of adiposity outcomes during GLP‐1‐based therapy. Effect estimates are presented as standardized mean differences (Hedges' *g*) with 95% confidence intervals and were pooled using a random‐effects model with restricted maximum likelihood estimation and Hartung‐Knapp adjustment.

#### 3.4.2 Muscle Quantity

Two studies contributed to the primary comparative analysis of muscle quantity (Figure 3). The pooled estimate was associated with substantial heterogeneity and very wide confidence intervals, precluding definitive conclusions regarding the effects of GLP‐1‐based therapy on muscle quantity.

**FIGURE 3:**
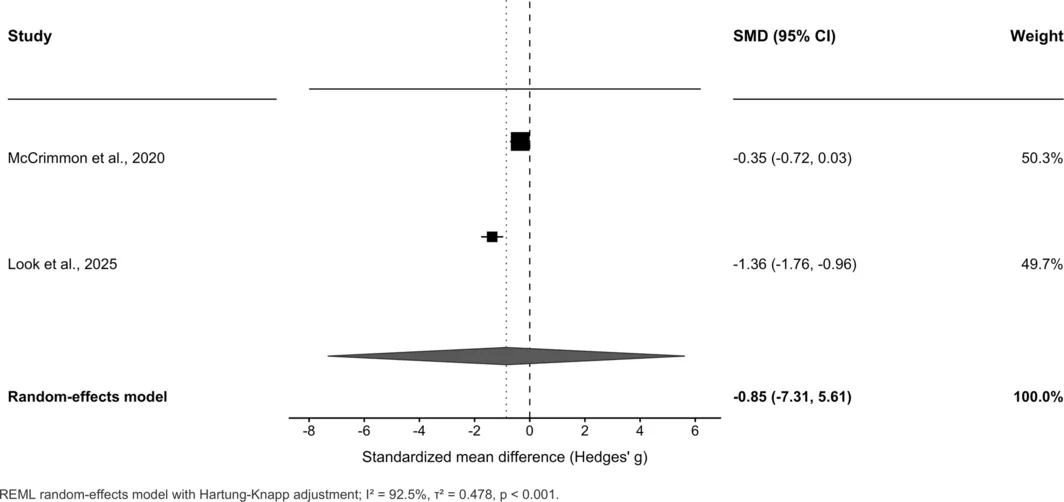
Forest plot of the primary comparative meta‐analysis of muscle quantity outcomes during GLP‐1‐based therapy. Effect estimates are presented as standardized mean differences (Hedges' *g*) with 95% confidence intervals and were pooled using a random‐effects model with restricted maximum likelihood estimation and Hartung‐Knapp adjustment.

#### 3.4.3 Narrative Evidence on Muscle Quality

Muscle quality outcomes were reported in comparative and exploratory imaging studies using heterogeneous methodologies (Table S8). Among the comparative studies, Pandey et al. [6] evaluated MRI‐derived thigh muscle fat and muscle volume *z* score, whereas Sattar et al. [21] assessed MRI‐derived intramuscular fat together with muscle volume measures. Exploratory studies, including Nelson et al. [33] and Volpe et al. [31], reported complementary imaging and functional markers of muscle composition, such as skeletal muscle attenuation, intermuscular adipose tissue, phase angle, and handgrip strength. Because these studies differed substantially in imaging modality, anatomical site, outcome definition, measurement scale, and variance reporting, quantitative synthesis was considered inappropriate. Accordingly, muscle quality outcomes were synthesized narratively.

### 3.5 Exploratory Single‐Arm Analyses

Exploratory single‐arm analyses consistently demonstrated reductions in adiposity and body weight (Figures S3 and S5), whereas changes in muscle quantity varied across studies (Figure S4). Because these analyses lacked comparator groups, they were considered hypothesis‐generating and were not interpreted as causal evidence.

### 3.6 Certainty of Evidence

We assessed the certainty of evidence for the primary comparative outcomes using the GRADE framework. The certainty of evidence was rated as low for the primary comparative adiposity outcome and very low for the primary comparative muscle quantity outcome (Table S4). The certainty for adiposity was downgraded because of substantial between‐study inconsistency and serious imprecision resulting from the limited number of comparative studies and wide confidence intervals. For muscle quantity, certainty was further downgraded because of concerns regarding risk of bias, substantial heterogeneity, serious imprecision, and the limited number of available comparative studies. Publication bias could not be formally assessed because of the small number of studies included in each meta‐analysis.

## 4 Discussion

GLP‐1‐based therapies were associated with body composition remodelling characterized by reductions in adiposity, whereas evidence regarding muscle quantity remained limited and heterogeneous and evidence addressing muscle quality was insufficient for quantitative synthesis. By applying a predefined physiological framework and restricting primary analyses to one physiologically coherent outcome per study and domain, we sought to provide a more coherent assessment of tissue remodelling during incretin‐based therapy. Although the available comparative studies generally favoured greater reductions in adiposity, only three studies contributed to the pooled analysis, which was characterized by substantial between‐study heterogeneity and wide confidence intervals. Together with the low certainty of evidence for adiposity and very low certainty for muscle quantity, these limitations preclude definitive conclusions regarding preferential fat loss or the effects of these therapies on skeletal muscle health. Importantly, the available muscle quantity outcomes were derived predominantly from lean tissue–based proxy measures and therefore should not be interpreted as direct assessments of skeletal muscle health or sarcopenia.

Our findings are consistent with accumulating evidence indicating that the weight loss achieved with GLP‐1 receptor agonists and dual incretin therapies predominantly reflects reductions in fat mass. In STEP‐1, semaglutide produced substantial decreases in body weight and total fat mass, with relative preservation of lean mass proportion despite reductions in absolute lean tissue [1]. Similarly, tirzepatide induced profound weight loss in SURMOUNT‐1 [2], and subsequent body composition analyses demonstrated that subsequent body composition analyses suggested that much of the observed weight loss reflected preferential depletion of adipose tissue [7]. These observations are compatible with the concept that incretin‐based therapies may promote tissue redistribution rather than uniform depletion of all body compartments.

Interpretation of lean mass changes deserves particular caution. Lean mass measurements derived from DXA do not specifically quantify skeletal muscle tissue and encompass several non‐contractile components, including body water, connective tissue, organ mass, and extracellular fluid [4]. Consequently, reductions in lean mass cannot be equated with skeletal muscle wasting or sarcopenia. Weight loss itself is accompanied by physiological adaptations involving reductions in body water and supporting tissues, and similar changes occur after lifestyle interventions and bariatric surgery [35]. Therefore, decreases in lean mass observed during GLP‐1‐based therapy should be interpreted within the broader context of whole‐body remodelling rather than viewed as evidence of detrimental muscle loss.

Distinguishing muscle quantity from muscle quality is particularly important when interpreting body composition changes during GLP‐1‐based therapy. Muscle quality is a multidimensional construct that reflects structural and compositional characteristics of skeletal muscle beyond its absolute quantity. The measures included in this review—including intramuscular fat infiltration, intermuscular adipose tissue, skeletal muscle attenuation, skeletal muscle density, phase angle, and handgrip strength—capture different biological domains of muscle composition, tissue integrity, and function and therefore should not be considered biologically interchangeable. Emerging evidence suggests that obesity is characterized not only by excess adiposity but also by ectopic fat accumulation within skeletal muscle, leading to impaired muscle composition and reduced contractile efficiency [36]. Accordingly, improvements in muscle quality may occur even when absolute lean mass decreases. Heise et al. reported reductions in muscle fat infiltration during tirzepatide treatment, indicating changes in tissue composition despite concomitant reductions in lean tissue mass [7]. Similarly, Pandey et al. demonstrated reductions in intermuscular adipose tissue and improvements in muscle composition parameters among patients with obesity‐related HFpEF receiving semaglutide [6]. Together, these findings suggest that body composition changes induced by incretin‐based therapies may involve qualitative improvements in skeletal muscle architecture that are not captured by conventional lean mass measurements.

Current concepts in sarcopenia further reinforce this interpretation. Contemporary definitions emphasize that muscle strength and physical performance have greater clinical relevance than isolated measures of lean mass [4, 5]. Accordingly, low appendicular lean mass alone is insufficient to establish the presence of sarcopenia. None of the available studies included in our review comprehensively assessed muscle strength, physical performance, and muscle quality simultaneously. Therefore, current evidence does not support the assumption that reductions in lean mass associated with GLP‐1‐based therapies represent treatment‐induced sarcopenia. However, the available evidence is also insufficient to exclude clinically relevant muscle loss in selected high‐risk populations, particularly because direct assessments of muscle quality, strength, and physical performance remain scarce. Rather, the available data highlight the limitations of relying exclusively on DXA‐derived lean tissue as a surrogate for skeletal muscle health.

The substantial between‐study heterogeneity observed in the comparative analyses likely reflects several sources of clinical and methodological variability. Included studies enrolled populations with obesity, Type 2 diabetes, obesity‐related HFpEF, and NAFLD, conditions that differ in baseline adiposity, metabolic dysfunction, and skeletal muscle characteristics. In addition, the evaluated therapies included liraglutide, semaglutide, tirzepatide, and retatrutide, which differ in pharmacological profile, weight‐loss efficacy, and mechanisms of action [3]. Treatment duration also varied considerably across studies, ranging from short‐term interventions to trials exceeding 1 year, which may influence the magnitude and temporal pattern of body composition remodelling. Finally, differences in body composition assessment methods and outcome definitions across studies, as summarized in Table 1 and Tables S7 and S8, likely contributed further to the observed heterogeneity.

From a biological perspective, the available findings are consistent with the hypothesis that incretin‐based therapies promote adipose tissue remodelling; however, the current evidence is insufficient to establish preferential reductions in adiposity or to exclude clinically relevant muscle loss in selected high‐risk populations. Adipose tissue is increasingly recognized as an active endocrine organ that contributes to systemic inflammation, insulin resistance, and ectopic lipid accumulation [37]. Reductions in visceral and ectopic fat depots may improve metabolic homeostasis and potentially favour skeletal muscle composition through decreased lipotoxicity and inflammation [36]. Accordingly, the observed changes in body composition should not be interpreted simply as weight loss, but rather as a process of tissue redistribution in which reductions in adiposity may contribute to improved metabolic and functional profiles. Future studies incorporating advanced imaging techniques, molecular markers, and standardized assessments of muscle function are needed to clarify the mechanisms underlying adipose‐muscle interactions during incretin‐based therapy.

These observations also have important clinical implications. Although current evidence does not support the notion that GLP‐1‐based therapies necessarily induce sarcopenia, preservation of musculoskeletal health should remain an integral component of obesity management. Current obesity and sarcopenia guidelines emphasize the importance of combining pharmacological treatment with resistance exercise, adequate dietary protein intake, and maintenance of physical function [5, 38]. Such multimodal approaches may optimize fat loss while preserving muscle mass, strength, and functional capacity. Future trials should incorporate standardized assessments of muscle strength and physical performance to determine whether tissue remodelling induced by incretin‐based therapies translates into clinically meaningful improvements in musculoskeletal health.

This study has several strengths. We implemented a physiological framework that separated adiposity, muscle quantity, and muscle quality, thereby minimizing conceptual overlap among outcomes. We restricted primary analyses to one physiologically coherent outcome per study and domain, reducing duplication and preserving statistical independence. Methodologically, we used restricted maximum likelihood estimation with Hartung‐Knapp adjustment, complemented by sensitivity analyses and GRADE assessment. Furthermore, integration of mechanistic studies provided additional context regarding tissue‐specific remodelling beyond conventional lean mass measurements.

This review has several limitations. First, the number of comparative studies available for quantitative synthesis was small, particularly for muscle quantity outcomes, resulting in imprecise pooled estimates and limited certainty of evidence. Second, substantial clinical and methodological heterogeneity persisted across studies with respect to participant characteristics, comparator interventions, imaging modalities, treatment duration, and outcome definitions. In particular, comparative studies used different control interventions, including placebo and active comparators, which may have influenced the magnitude and interpretation of standardized mean differences. Third, most studies were not specifically designed to evaluate skeletal muscle remodelling, and standardized assessments of muscle strength, physical performance, and muscle quality were infrequently reported. Consequently, current evidence remains insufficient to determine whether incretin‐based therapies exert beneficial, neutral, or unfavourable effects on skeletal muscle quantity or overall muscle health.

In conclusion, current comparative evidence suggests greater reductions in adiposity during GLP‐1‐based therapy; however, the limited number of available comparative studies, substantial between‐study heterogeneity, and imprecision of the pooled estimates preclude definitive conclusions regarding preferential fat loss or preservation of skeletal muscle health. Current evidence does not support the assumption that reductions in lean mass necessarily represent impaired muscle health or treatment‐induced sarcopenia, although direct assessments of muscle quality, muscle strength, and physical performance remain scarce. Future adequately powered comparative studies incorporating standardized assessments of muscle quantity, muscle quality, muscle strength, and physical performance are required to clarify the long‐term musculoskeletal effects of incretin‐based therapy.

## Funding

Alberto Sá Filho was supported by the Brazilian National Council for Scientific and Technological Development (Conselho Nacional de Desenvolvimento Científico e Tecnológico—CNPq) through a Research Productivity Fellowship (grant number 300819/2026‐9).

## Conflicts of Interest

The authors declare no conflicts of interest.

## Supporting information


**Figure S1:** Risk‐of‐bias assessment of randomized controlled trials using the Cochrane Risk of Bias 2 (RoB 2) tool.
**Figure S2:** Risk‐of‐bias assessment of non‐randomized studies using the Risk Of Bias In Non‐randomized Studies of Interventions (ROBINS‐I) tool.
**Figure S3:** Exploratory single‐arm analysis of adiposity outcomes.
**Figure S4:** Exploratory single‐arm analysis of muscle quantity outcomes.
**Figure S5:** Exploratory single‐arm analysis of body weight outcomes.
**Figure S6:** Narrative framework for muscle quality outcomes and imaging‐based evidence.
**Figure S7:** Leave‐one‐out sensitivity analysis for the primary comparative adiposity meta‐analysis. Sequential omission of individual studies was performed as a supportive diagnostic procedure to explore the influence of individual studies on the pooled estimate. Because only three comparative studies contributed to the primary analysis, these results should be interpreted descriptively and not as formal evidence of robustness.
**Figure S8:** Baujat plot for the primary comparative adiposity meta‐analysis. The Baujat plot was used as a supportive diagnostic procedure to identify the relative contribution of individual studies to heterogeneity and overall influence. Given the limited number of comparative studies, this diagnostic should be interpreted descriptively and not as formal evidence of robustness.
**Table S1:** Expanded study characteristics.
**Table S2:** Complete search strategies.
**Table S3:** Detailed risk‐of‐bias assessments.
**Table S4:** GRADE certainty assessment for primary comparative outcomes.
**Table S5:** Outcome classification framework and analytical hierarchy.
**Table S6:** Leave‐one‐out estimates for the primary comparative adiposity analysis.
**Table S7:** Study‐level implementation of the predefined analytical hierarchy and outcome selection for quantitative synthesis.
**Table S8:** Structured narrative synthesis of muscle quality outcomes and reasons for non‐pooling.

## Data Availability

The data that support the findings of this study are available from the corresponding author upon reasonable request. All data analyzed in this systematic review and meta‐analysis were extracted from published studies, and the sources of these data are fully cited within the article and its Supplementary Materials. No new individual participant data were generated during this study.
