# Supplementary material for: Body Composition Remodelling During GLP‐1‐Based Therapy: A Systematic Review and Meta‐Analysis Using a Hierarchical Physiological Framework
Source: Diabetes Obes Metab. 2026 Aug 17;28(11):9849–60. doi: 10.1111/dom.71220 (PMC13646380; doi:10.1111/dom.71220)
Supplement: Supplementary file 1 [file DOM-28-9849-s001.docx]

**SUPPLEMENTARY MATERIAL**

Maria Luizza Lopes^1,2^, MD, MSc; Maria Eduarda G. Chiappa^3^, Rodrigo Fuentes Kloss^4^, PT, MSc; Marcelo Tuesta^5^, PT, PhD; Paula Santos^6^, PT, MSc; Alberto Souza Sá Filho^6^, PE, PhD; Gaspar R. Chiappa^6,7^, PT, PhD

^1^Secretariat of Health of the Federal District, Brasília, Brazil.

^2^School of Public Health of the Federal District, Brasília, Brazil.

^3^Federal University of Rio Grande do Sul, Porto Alegre, Brazil.

^4^Health Sciences School, Faculty of Medicine, Pontificia Universidad Católica de Chile, Santiago, Chile.

^5^Exercise and Rehabilitation Sciences Institute, School of Physical Therapy, Faculty of Rehabilitation Sciences, Universidad Andres Bello, Santiago 7591538, Chile.

^6^Graduate Program in Human Movement and Rehabilitation of Evangelical University of Goiás, Brazil.

^7^Faculty of Health Sciences, Universidad Autónoma de Chile, Providencia, Santiago, Chile.

**Running title**

**GLP-1 Therapy and Body Composition**

**Supplementary Figures**

**Figure S1.** Risk-of-bias assessment of randomized controlled trials using the Cochrane Risk of Bias 2 (RoB 2) tool.

**Figure S2.** Risk-of-bias assessment of non-randomized studies using the Risk Of Bias In Non-randomized Studies of Interventions (ROBINS-I) tool.

**Figure S3.** Exploratory single-arm meta-analysis of adiposity outcomes during GLP-1-based therapy.

**Figure S4.** Exploratory single-arm meta-analysis of muscle quantity outcomes during GLP-1-based therapy.

**Figure S5.** Exploratory single-arm meta-analysis of body weight outcomes during GLP-1-based therapy.

**Figure S6.** Narrative synthesis of muscle quality outcomes and imaging-based evidence.

**Figure S7.** Leave-one-out sensitivity analysis for the primary comparative adiposity meta-analysis. Sequential omission of individual studies was performed as a **supportive diagnostic procedure** to explore the influence of individual studies on the pooled estimate. Because only three comparative studies contributed to the primary analysis, these results should be interpreted descriptively and not as formal evidence of robustness.

**Figure S8.** **Baujat plot for the primary comparative adiposity meta-analysis.** The Baujat plot was used as a **supportive diagnostic procedure** to identify the relative contribution of individual studies to heterogeneity and overall influence. Given the limited number of comparative studies, this diagnostic should be interpreted descriptively and not as formal evidence of robustness.

**Supplementary Tables**

**Table S1.** Expanded study characteristics.

**Table S2.** Complete search strategies.

**Table S3.** Detailed risk-of-bias assessments.

**Table S4.** GRADE certainty assessment for primary comparative outcomes.

**Table S5.** Outcome classification framework and analytical hierarchy.

**Table S6.** Leave-one-out estimates for the primary comparative adiposity analysis.

**Table S7.** Study-level implementation of the predefined analytical hierarchy and outcome selection for quantitative synthesis.

**Table S8.** Structured narrative synthesis of muscle quality outcomes and reasons for non-pooling

**SUPPLEMENTARY FIGURES**

**Figure S1 and S2.** Risk-of-bias assessment of randomized controlled trials using RoB 2 and non-randomized studies using ROBINS-I.

**Figure S3.** Exploratory single-arm analysis of adiposity outcomes.

**Figure S4.** Exploratory single-arm analysis of muscle quantity outcomes.

**Figure S5.** Exploratory single-arm analysis of body weight outcomes.

**Figure S6.** Narrative framework for muscle quality outcomes and imaging-based evidence.

**Figure S7.** **Leave-one-out sensitivity analysis for the primary comparative adiposity meta-analysis.** Sequential omission of individual studies was performed as a **supportive diagnostic procedure** to explore the influence of individual studies on the pooled estimate. Because only three comparative studies contributed to the primary analysis, these results should be interpreted descriptively and not as formal evidence of robustness.

**Figure S8.** **Baujat plot for the primary comparative adiposity meta-analysis.** The Baujat plot was used as a **supportive diagnostic procedure** to identify the relative contribution of individual studies to heterogeneity and overall influence. Given the limited number of comparative studies, this diagnostic should be interpreted descriptively and not as formal evidence of robustness.

Table S1. Expanded study characteristics

| **Study** | **Design** | **Population** | **Intervention** | **Comparator** | **Body composition method** | **Analytical role** |
| --- | --- | --- | --- | --- | --- | --- |
| Coskun et al., 2025 | Phase 2 randomized controlled trial, DXA substudy | Adults with type 2 diabetes | Retatrutide | Placebo or dulaglutide | DXA | Primary comparative adiposity |
| Look et al., 2025 | SURMOUNT-1 randomized controlled trial, DXA substudy | Adults with obesity or overweight without diabetes | Tirzepatide | Placebo | DXA | Primary comparative adiposity and muscle quantity |
| McCrimmon et al., 2020 | Randomized controlled trial, DXA substudy | Adults with type 2 diabetes receiving metformin | Semaglutide | Canagliflozin | DXA | Primary comparative adiposity and muscle quantity |
| Pandey et al., 2024 | Prespecified secondary analysis of randomized controlled trial | Adults with overweight or obesity without diabetes | Liraglutide plus lifestyle intervention | Placebo plus lifestyle intervention | MRI | Narrative muscle quality evidence |
| Feng et al., 2019 | Randomized open-label active-comparator trial | Adults with type 2 diabetes and NAFLD | Liraglutide | Metformin or gliclazide | DXA | Comparative supportive evidence |
| Heise et al., 2023 | Phase 1 randomized placebo-controlled trial | Adults with type 2 diabetes | Tirzepatide | Placebo | MRI/body composition assessment | Supportive narrative evidence |
| Sattar et al., 2025 | Post hoc analysis of phase 3 randomized trial MRI substudy | Adults with type 2 diabetes and overweight or obesity | Tirzepatide | Insulin degludec | MRI | Supportive imaging evidence |
| Yang et al., 2025 | Retrospective real-world comparative cohort | Adults with type 2 diabetes and chronic kidney disease | SGLT2 inhibitor plus semaglutide | SGLT2 inhibitor alone | Body composition assessment | Observational supportive evidence |
| Basolo et al., 2025 | Prospective single-arm study | Adults with obesity | Liraglutide | None | DXA | Exploratory single-arm analysis |
| Bhatti et al., 2025 | Retrospective real-world cohort | Adults with overweight or obesity in multidisciplinary weight management | Semaglutide or tirzepatide plus lifestyle programme | None | BIA | Exploratory single-arm analysis |
| Chun et al., 2025 | Retrospective single-arm real-world cohort | Adults in commercial wellness or weight management setting | Compounded semaglutide/cyanocobalamin | None | BIA | Exploratory single-arm analysis |
| Gjersdal et al., 2025 | Case series/single-arm longitudinal study | Adult women with hypothalamic obesity after craniopharyngioma treatment | Semaglutide | None | Body composition assessment | Narrative exploratory evidence |
| Ishikawa et al., 2024 | Single-arm add-on cohort | Adults with type 2 diabetes-associated NAFLD/NASH | Oral semaglutide added to luseogliflozin | Pre-post comparison after SGLT2 inhibitor run-in | SMF-BIA | Exploratory single-arm analysis |
| Nelson et al., 2024 | Retrospective intrapatient CT cohort | Adults using semaglutide with pre-treatment and post-treatment CT imaging | Semaglutide | None | CT | Exploratory single-arm secondary/supportive muscle quality relevance |
| Rodriguez Jimenez et al., 2024 | Retrospective quasi-experimental study | Adults with type 2 diabetes and obesity | Subcutaneous or oral semaglutide | None | BIA | Exploratory single-arm analysis |
| Uchiyama et al., 2023 | Retrospective single-arm longitudinal study | Japanese adults with type 2 diabetes | Oral semaglutide | None | BIA | Exploratory single-arm analysis |
| Volpe et al., 2022 | Prospective single-arm real-life study | Adults with type 2 diabetes | Once-weekly subcutaneous semaglutide | None | BIA/body composition platform | Exploratory single-arm and narrative functional evidence |
| Volpe et al., 2023 | Prospective single-arm real-life study | Adults with type 2 diabetes | Oral semaglutide | None | BIA/body composition platform | Exploratory single-arm secondary/supportive muscle quality relevance |
| Xiang et al., 2023 | Retrospective real-world single-arm cohort | Chinese adults with obesity without diabetes | Semaglutide plus lifestyle intervention | None | BIA | Exploratory single-arm analysis |

Abbreviations: BIA, bioelectrical impedance analysis; CT, computed tomography; DXA, dual-energy X-ray absorptiometry; MRI, magnetic resonance imaging; NAFLD, non-alcoholic fatty liver disease; NASH, non-alcoholic steatohepatitis; SGLT2, sodium-glucose cotransporter 2; SMF-BIA, segmental multifrequency bioelectrical impedance analysis.

**Table S2. Complete search strategies.**

| Database | Search strategy |
| --- | --- |
| PubMed/MEDLINE | (“Glucagon-Like Peptide 1 Receptor Agonists”[Mesh] OR “glucagon-like peptide-1 receptor agonist*” OR GLP-1 OR GLP-1RA OR semaglutide OR tirzepatide OR liraglutide OR dulaglutide OR exenatide OR lixisenatide OR albiglutide OR retatrutide OR incretin therapy OR dual incretin therapy) AND (“Body Composition”[Mesh] OR body composition OR adiposity OR fat mass OR body fat OR lean mass OR lean body mass OR fat-free mass OR skeletal muscle OR muscle mass OR muscle quantity OR muscle quality OR intramuscular fat OR intermuscular fat OR myosteatosis OR muscle attenuation OR phase angle OR DXA OR DEXA OR “dual-energy X-ray absorptiometry” OR MRI OR “magnetic resonance imaging” OR CT OR “computed tomography” OR “bioelectrical impedance” OR BIA) AND (obesity OR overweight OR “type 2 diabetes” OR T2D OR T2DM OR metabolic disease OR cardiometabolic disease) |
| Embase | (‘glucagon like peptide 1 receptor agonist’/exp OR ’glucagon-like peptide-1 receptor agonist*’ OR glp-1 OR glp-1ra OR semaglutide OR tirzepatide OR liraglutide OR dulaglutide OR exenatide OR lixisenatide OR albiglutide OR retatrutide OR ‘incretin therapy’ OR ‘dual incretin therapy’) AND (‘body composition’/exp OR ‘body composition’ OR adiposity OR ‘fat mass’ OR ‘body fat’ OR ‘lean mass’ OR ‘lean body mass’ OR ‘fat free mass’ OR ‘skeletal muscle’ OR ‘muscle mass’ OR ‘muscle quantity’ OR ‘muscle quality’ OR ‘intramuscular fat’ OR ‘intermuscular fat’ OR myosteatosis OR ‘muscle attenuation’ OR ‘phase angle’ OR dxa OR dexa OR ‘dual energy x ray absorptiometry’ OR mri OR ‘magnetic resonance imaging’ OR ct OR ‘computed tomography’ OR ‘bioelectrical impedance’ OR bia) AND (obesity OR overweight OR ‘type 2 diabetes’ OR t2d OR t2dm OR ‘metabolic disease’ OR ‘cardiometabolic disease’) |
| Cochrane CENTRAL | (“glucagon-like peptide-1 receptor agonist*” OR GLP-1 OR GLP-1RA OR semaglutide OR tirzepatide OR liraglutide OR dulaglutide OR exenatide OR lixisenatide OR albiglutide OR retatrutide OR incretin therapy OR dual incretin therapy) AND (“body composition” OR adiposity OR “fat mass” OR “body fat” OR “lean mass” OR “lean body mass” OR “fat-free mass” OR “skeletal muscle” OR “muscle mass” OR “muscle quantity” OR “muscle quality” OR “intramuscular fat” OR “intermuscular fat” OR myosteatosis OR “muscle attenuation” OR “phase angle” OR DXA OR DEXA OR “dual-energy X-ray absorptiometry” OR MRI OR “magnetic resonance imaging” OR CT OR “computed tomography” OR “bioelectrical impedance” OR BIA) AND (obesity OR overweight OR “type 2 diabetes” OR T2D OR T2DM OR “metabolic disease” OR “cardiometabolic disease”) |

**Search date:** Searches were conducted from database inception through May 2026.

**Additional sources:** Reference lists of eligible studies and relevant reviews were manually screened to identify additional records.

**Restrictions:** No language restrictions were applied during the search.

**Table S3. Detailed risk-of-bias assessments.**

| **Study** | **Study design** | **Tool** | **Domain-level concerns** | **Overall judgement** | **Main reason for judgement** | **Use in analysis** |
| --- | --- | --- | --- | --- | --- | --- |
| Coskun et al., 2025 | Phase 2 RCT, DXA substudy | RoB 2 | Some concerns for randomization, deviations, missing outcome data, and selective reporting; low risk for outcome measurement | Some concerns | DXA substudy within a multi-arm phase 2 trial; body composition outcome reporting and missing DXA data required caution | Primary comparative adiposity |
| Look et al., 2025 | SURMOUNT-1 RCT, DXA substudy | RoB 2 | Some concerns for missing outcome data and selective reporting; low risk for randomization, deviations, and outcome measurement | Some concerns | Randomized parent trial, but substudy reporting and missing DXA data introduced uncertainty | Primary comparative adiposity and muscle quantity |
| McCrimmon et al., 2020 | RCT, DXA substudy | RoB 2 | Some concerns for deviations, missing outcome data, and selective reporting; low risk for randomization and outcome measurement | Some concerns | Objective DXA outcome assessment, but substudy design and attrition/reporting required caution | Primary comparative adiposity and muscle quantity |
| Pandey et al., 2024 | Prespecified secondary analysis of RCT | RoB 2 | Some concerns for missing outcome data and selective reporting; low risk for randomization, deviations, and outcome measurement | Some concerns | Randomized parent trial, but MRI-complete secondary analysis required cautious interpretation | Narrative muscle quality evidence |
| Feng et al., 2019 | Randomized open-label active-comparator trial | RoB 2 | Some concerns for randomization, deviations, missing outcome data, and selective reporting; low risk for outcome measurement | Some concerns | Open-label design, small sample, active comparator, and imputed change SDs reduced certainty | Supportive comparative evidence |
| Heise et al., 2023 | Phase 1 randomized placebo-controlled trial | RoB 2 | Some concerns for randomization, deviations, missing outcome data, and selective reporting; low risk for outcome measurement | Some concerns | Mechanistic phase 1 trial; incomplete variance reporting prevented quantitative synthesis | Supportive narrative evidence |
| Sattar et al., 2025 | Post hoc analysis of phase 3 RCT MRI substudy | RoB 2 | Some concerns for deviations and selective reporting; low risk for randomization, missing outcome data, and outcome measurement | Some concerns | Randomized parent trial but open-label/post hoc MRI substudy and selective reporting concerns | Supportive imaging evidence |
| Yang et al., 2025 | Retrospective real-world comparative cohort | ROBINS-I | Serious selection and confounding concerns; moderate concerns for missing data, outcome measurement, and reporting | Serious | Nonrandomized comparison with confounding by indication and combination therapy | Observational supportive evidence |
| Basolo et al., 2025 | Prospective single-arm study | ROBINS-I | Serious confounding concerns; moderate selection, missing data, and reporting concerns; low concern for outcome measurement | Serious | No control group, small sample, and cointerventions limited causal attribution | Exploratory single-arm analysis |
| Bhatti et al., 2025 | Retrospective real-world cohort | ROBINS-I | Serious selection, confounding, and missing data concerns; moderate outcome measurement and reporting concerns | Serious | No control group, retrospective design, BIA measurement, follow-up attrition, and lifestyle cointervention | Exploratory single-arm analysis |
| Chun et al., 2025 | Retrospective single-arm real-world cohort | ROBINS-I | Serious selection and confounding concerns; moderate missing data, outcome measurement, and reporting concerns | Serious | No control group, commercial setting, compounded formulation, BIA assessment, and short follow-up | Exploratory single-arm analysis |
| Gjersdal et al., 2025 | Case series/single-arm longitudinal study | ROBINS-I | Critical selection and confounding concerns; serious missing data and reporting concerns; moderate outcome measurement concerns | Critical | Very small case series in a highly selected rare population without comparator | Narrative exploratory evidence |
| Ishikawa et al., 2024 | Single-arm add-on cohort | ROBINS-I | Serious selection and confounding concerns; moderate missing data, outcome measurement, and reporting concerns | Serious | Add-on design after SGLT2 inhibitor run-in limited attribution to semaglutide | Exploratory single-arm analysis |
| Nelson et al., 2024 | Retrospective intrapatient CT cohort | ROBINS-I | Serious selection, confounding, and missing data concerns; low outcome measurement concern; moderate reporting concern | Serious | Opportunistic CT selection, no comparator, variable treatment timing, and incomplete treatment exposure | Exploratory single-arm secondary/supportive muscle quality relevance |
| Rodriguez Jimenez et al., 2024 | Retrospective quasi-experimental study | ROBINS-I | Serious selection and confounding concerns; moderate missing data, outcome measurement, and reporting concerns | Serious | No non-GLP-1 comparator and BIA-based measurements limited causal interpretation | Exploratory single-arm analysis |
| Uchiyama et al., 2023 | Retrospective single-arm longitudinal study | ROBINS-I | Serious confounding concerns; moderate selection, missing data, outcome measurement, and reporting concerns | Serious | No control group, small sample, BIA assessment, and unmeasured diet/exercise cointerventions | Exploratory single-arm analysis |
| Volpe et al., 2022 | Prospective single-arm real-life study | ROBINS-I | Serious confounding concerns; moderate selection, missing data, outcome measurement, and reporting concerns | Serious | Prospective design reduced some concerns, but absence of comparator and real-life cointerventions limited causal inference | Exploratory single-arm and narrative functional evidence |
| Volpe et al., 2023 | Prospective single-arm real-life study | ROBINS-I | Serious confounding concerns; moderate selection, missing data, outcome measurement, and reporting concerns | Serious | Prospective but uncontrolled design, BIA assessment, and assumptions for variance estimates limited certainty | Exploratory single-arm secondary/supportive muscle quality relevance |
| Xiang et al., 2023 | Retrospective real-world single-arm cohort | ROBINS-I | Serious selection and confounding concerns; moderate missing data, outcome measurement, and reporting concerns | Serious | No control group and lifestyle intervention limited attribution to semaglutide-specific effects | Exploratory single-arm analysis |

Abbreviations: BIA, bioelectrical impedance analysis; CT, computed tomography; DXA, dual-energy X-ray absorptiometry; GLP-1, glucagon-like peptide-1; MRI, magnetic resonance imaging; RCT, randomized controlled trial; RoB 2, revised Cochrane Risk of Bias tool for randomized trials; ROBINS-I, Risk Of Bias In Non-randomized Studies of Interventions; SD, standard deviation; SGLT2, sodium-glucose cotransporter 2.

**Table S4. GRADE certainty assessment for primary comparative outcomes**

| **Outcome** | **Studies (n)** | **Participants (n)** | **Pooled effect, Hedges’ g (95% CI)** | **Heterogeneity** | **Risk of bias** | **Inconsistency** | **Indirectness** | **Imprecision** | **Publication bias** | **Certainty of evidence** | **Explicit reasons for downgrading** |
| --- | --- | --- | --- | --- | --- | --- | --- | --- | --- | --- | --- |
| **Primary comparative adiposity outcome** | 3 | 369 | −1.30 (−3.74 to 1.13) | I² = 94.2%; τ² = 0.902; p < 0.001 | Not serious | Serious | Not serious | Serious | Not assessed | **Low ⨁⨁◯◯** | The evidence was downgraded one level for **inconsistency**because of substantial between-study heterogeneity (I² = 94.2%) and marked variation in effect magnitude. It was downgraded one level for **imprecision**because only three studies contributed to the analysis and the 95% confidence interval was wide and crossed the null. Risk of bias was not considered serious because the contributing studies were randomized comparative trials with overall RoB 2 judgements of some concerns, without evidence that these concerns substantially altered the direction of the pooled adiposity estimate. Publication bias could not be assessed because fewer than 10 studies were available. |
| **Primary comparative lean tissue/muscle quantity proxy outcome** | 2 | 297 | −0.85 (−7.31 to 5.61) | I² = 92.5%; τ² = 0.478; p < 0.001 | Not serious | Serious | Serious | Serious | Not assessed | **Very low ⨁◯◯◯** | The evidence was downgraded one level for **inconsistency**because of substantial heterogeneity (I² = 92.5%) and materially different study-level effects. It was downgraded one level for **indirectness**because the available outcomes were DXA-derived total lean mass or lean tissue measures, which are proxy measures and do not directly assess contractile skeletal muscle, muscle quality, strength, physical performance, or sarcopenia. It was downgraded one level for **imprecision**because only two studies contributed to the analysis and the 95% confidence interval was extremely wide and crossed the null. Risk of bias was not considered serious because both contributing studies were randomized comparative trials with overall RoB 2 judgements of some concerns; the additional uncertainty relates primarily to outcome indirectness rather than study-level bias. Publication bias could not be assessed because fewer than 10 studies were available. |

**GRADE**, Grading of Recommendations Assessment, Development and Evaluation; **CI**, confidence interval; **DXA**, dual-energy X-ray absorptiometry; **RoB 2**, revised Cochrane Risk of Bias tool for randomized trials; **τ²**, estimated between-study variance. Negative Hedges’ g values indicate a greater reduction in the GLP-1-based therapy group than in the comparator group. For adiposity, a negative value represents a greater reduction in adiposity. For the lean tissue/muscle quantity proxy outcome, a negative value represents a greater reduction in lean tissue and should not be interpreted as evidence of impaired muscle health or sarcopenia. Participant totals were calculated from the analysis-specific intervention and comparator samples: adiposity, Coskun et al. (48/24), Look et al. (79/40), and McCrimmon et al. (88/90); muscle quantity proxy, Look et al. (79/40) and McCrimmon et al. (88/90). The pooled estimates and heterogeneity statistics correspond to Figures 2 and 3.

**Table S5. Outcome classification framework and analytical hierarchy.**

| **Domain** | **Outcomes included** | **Preferred outcome for primary analysis** | **Outcomes not pooled in the primary analysis** | **Analytical role** | **Rationale** |
| --- | --- | --- | --- | --- | --- |
| Adiposity | Total fat mass, fat mass, fat mass index, body fat percentage | Whole-body fat mass assessed by DXA or equivalent whole-body method | Visceral adipose tissue, trunk fat, android fat, regional adiposity, liver fat | Primary comparative analysis and exploratory single-arm analysis | Whole-body fat mass provides the most direct estimate of adiposity and avoids mixing regional fat depots with total adiposity measures. |
| Muscle quantity | Total lean mass, lean soft tissue, fat-free mass, skeletal muscle mass estimates | Whole-body lean mass or total lean tissue assessed by DXA or equivalent whole-body method | Appendicular-only lean mass, isolated thigh muscle volume, regional skeletal muscle estimates | Primary comparative analysis and exploratory single-arm analysis | Lean tissue estimates are commonly used as surrogate measures of muscle quantity but do not directly assess skeletal muscle integrity, composition, strength, or function. |
| Muscle quality | Intramuscular fat infiltration, muscle attenuation, muscle density, phase angle, MRI-derived muscle fat | Imaging-derived intramuscular fat or muscle composition measures | Lean mass, fat-free mass, body weight, anthropometric measures | Narrative synthesis only | Muscle quality reflects tissue composition and metabolic characteristics and should remain analytically distinct from muscle quantity. Quantitative synthesis was not performed because of limited data, methodological heterogeneity, and unavailable variance estimates. |
| Body weight | Body weight change, percentage body weight change, BMI change | Body weight change | Fat mass, lean mass, muscle quality measures | Exploratory supportive analysis | Body weight is clinically relevant but cannot distinguish adipose tissue loss from lean tissue or muscle-related changes. |

**Footnote:** When multiple eligible outcomes were available within the same study and domain, one physiologically coherent outcome was selected according to the prespecified hierarchy to avoid double counting. Primary comparative analyses prioritized whole-body measures and comparative designs. Exploratory single-arm analyses were used only to describe directionality and were not interpreted as causal evidence. Muscle quality outcomes were synthesized narratively because available data were sparse, methodologically heterogeneous, and incompletely reported.

**Table S6. Leave-one-out estimates for the primary comparative adiposity analysis.**

| **Omitted study** | **Pooled SMD (Hedges' g)** | **95% CI** | **I² (%)** |
| --- | --- | --- | --- |
| McCrimmon et al., 2020 | -1.79 | -4.23 to 0.65 | 8.3 |
| Coskun et al., 2025 | -0.94 | -10.11 to 8.23 | 96.2 |
| Look et al., 2025 | -1.13 | -12.98 to 10.72 | 95.6 |

**Footnote:** Each row shows the pooled standardized mean difference after sequential omission of one study from the primary comparative adiposity meta-analysis. Effect estimates are presented as Hedges' g with 95% confidence intervals. Each row shows the pooled standardized mean difference after sequential omission of one study from the primary comparative adiposity analysis. SMD values are expressed as Hedges g

.

Table S7. Study-level implementation of the predefined analytical hierarchy and outcome selection for quantitative synthesis.

| **Study** | **Domain** | **Outcome selected for analysis** | **Unit** | **Primary analysis** | **Selection status** | **Variance source** | **Sample size (Intervention / Control)** | **Reason for selection** |
| --- | --- | --- | --- | --- | --- | --- | --- | --- |
| Coskun et al., 2025 | Adiposity | Total fat mass | kg | **Yes** | Primary comparative | Reported SD | 48 / 24 | Highest-ranked whole-body adiposity measure according to the prespecified hierarchy |
| Look et al., 2025 | Adiposity | Total fat mass | kg | **Yes** | Primary comparative | Reported SD | 79 / 40 | Highest-ranked whole-body adiposity measure |
| Look et al., 2025 | Muscle quantity | Total lean mass | kg | **Yes** | Primary comparative | Reported SD | 79 / 40 | Preferred whole-body muscle quantity proxy according to the prespecified hierarchy |
| McCrimmon et al., 2020 | Adiposity | Total fat mass | kg | **Yes** | Primary comparative | Reported SD | 88 / 90 | Highest-ranked whole-body adiposity measure |
| McCrimmon et al., 2020 | Muscle quantity | Total lean mass | kg | **Yes** | Primary comparative | Reported SD | 88 / 90 | Preferred whole-body muscle quantity proxy |
| Feng et al., 2019 | Adiposity | Total fat mass | kg | **No** | Supportive comparative | Imputed change SD | 20 / 20 | Included as supportive comparative evidence only; excluded from the primary pooled analysis according to the prespecified hierarchy |
| Pandey et al., 2024 | Muscle quality | Intermuscular fat (MRI) | Reported unit | **No** | Narrative synthesis | Reported | 57 / 57 | Muscle quality outcomes were not sufficiently homogeneous for quantitative pooling |
| Heise et al., 2023 | Muscle quality | Intramuscular fat (MRI) | MRI-derived measure | **No** | Narrative synthesis | Variance unavailable | 41 / 24 | Variance estimates were unavailable, precluding quantitative synthesis |
| Sattar et al., 2025 | Muscle quality | Muscle fat composition (MRI) | MRI-derived measure | **No** | Supportive imaging evidence | Reported | Original study sample | Imaging findings supported the narrative synthesis but were not quantitatively pooled because they were not directly comparable with the predefined muscle quality outcome |
| Basolo et al., 2025 | Adiposity | Total fat mass | kg | **No** | Exploratory single-arm | Reported SD | Original study sample | Included only in exploratory single-arm analyses |
| Bhatti et al., 2025 | Adiposity | Total fat mass | kg | **No** | Exploratory single-arm | Reported SD | Original study sample | Included only in exploratory single-arm analyses |
| Chun et al., 2025 | Adiposity | Total fat mass | kg | **No** | Exploratory single-arm | Reported SD | Original study sample | Included only in exploratory single-arm analyses |
| Gjersdal et al., 2025 | Body weight | Body weight | kg | **No** | Narrative exploratory | Reported | Original study sample | Rare population; descriptive evidence only |
| Ishikawa et al., 2024 | Muscle quantity | Total lean mass | kg | **No** | Exploratory single-arm | Reported SD | Original study sample | Included only in exploratory single-arm analyses |
| Nelson et al., 2024 | Muscle quality | CT-derived muscle composition | CT | **No** | Supportive imaging evidence | Reported | Original study sample | Supportive imaging evidence only |
| Rodriguez Jimenez et al., 2024 | Adiposity | Total fat mass | kg | **No** | Exploratory single-arm | Reported SD | Original study sample | Included only in exploratory single-arm analyses |
| Uchiyama et al., 2023 | Muscle quantity | Total lean mass | kg | **No** | Exploratory single-arm | Reported SD | Original study sample | Included only in exploratory single-arm analyses |
| Volpe et al., 2022 | Muscle quantity | Total lean mass | kg | **No** | Exploratory single-arm | Reported SD | Original study sample | Included only in exploratory single-arm analyses |
| Volpe et al., 2023 | Muscle quantity | Total lean mass | kg | **No** | Exploratory single-arm | Reported SD | Original study sample | Included only in exploratory single-arm analyses |
| Xiang et al., 2023 | Muscle quantity | Total lean mass | kg | **No** | Exploratory single-arm | Reported SD | Original study sample | Included only in exploratory single-arm analyses |

Only one physiologically coherent outcome per study and domain was selected for the primary comparative analyses to avoid double counting. Outcome selection followed the prespecified analytical hierarchy described in Table S5. The column "Primary analysis" indicates whether the selected outcome contributed to the primary comparative meta-analysis. "Selection status" defines the methodological role of each study within the review (primary comparative, supportive comparative, supportive imaging evidence, narrative synthesis, narrative exploratory, or exploratory single-arm analysis). Variance source indicates whether variability estimates were reported directly, converted, imputed, or unavailable.

Table S8. Structured narrative synthesis of muscle quality outcomes and reasons for non-pooling

| **Study** | **Study design and population** | **Intervention and comparator** | **Follow-up** | **Imaging modality** | **Muscle quality outcome(s)** | **Anatomical region** | **Principal finding** | **Variance reporting** | **Analytical role** | **Reason for narrative synthesis rather than quantitative pooling** |
| --- | --- | --- | --- | --- | --- | --- | --- | --- | --- | --- |
| **Pandey et al., 2024** | Prespecified secondary analysis of a randomized controlled trial involving adults with overweight or obesity | Liraglutide 3.0 mg plus lifestyle intervention versus placebo plus lifestyle intervention | Median 36 weeks | MRI | Thigh muscle fat and muscle composition; muscle volume z score was additionally reported as a muscle quantity measure | Thigh skeletal muscle | Treatment was associated with a reduction in thigh muscle fat, suggesting improvement in muscle composition. Muscle volume was evaluated separately as a muscle quantity proxy. | Variance reporting was insufficient or not provided in a form suitable for calculation of a standardized comparative effect for the muscle quality outcome | Narrative muscle quality evidence | The reported outcome represented an MRI-derived measure of thigh muscle fat that was not directly comparable with the intramuscular fat metrics used in the other imaging study. Differences in outcome definition, reporting scale, anatomical assessment, and availability of variance estimates prevented reliable quantitative pooling. |
| **Sattar et al., 2025** | Post hoc MRI substudy of a randomized phase 3 trial involving adults with type 2 diabetes and overweight or obesity | Tirzepatide, pooled doses, versus insulin degludec | 52 weeks | MRI | Intramuscular fat; muscle volume and muscle volume z score were also reported | Skeletal muscle region assessed in the MRI substudy | Tirzepatide was associated with reductions in intramuscular fat, providing supportive imaging evidence of changes in muscle composition. Muscle volume outcomes were considered separately from muscle quality. | Outcome reporting did not provide sufficiently compatible comparative variance data for inclusion with the Pandey et al. estimate | Supportive comparative imaging evidence | The study used a distinct intramuscular fat metric, comparator, population, treatment duration, and reporting format. The muscle quality outcome could not be harmonized with the thigh muscle fat measure reported by Pandey et al.; incomplete or non-comparable variance reporting further precluded pooling. |

Muscle quality was defined as imaging-based assessment of fat infiltration or composition within or around skeletal muscle and was considered conceptually distinct from muscle quantity. Muscle volume, lean mass, and related measurements were classified as muscle quantity proxies and were not interpreted as direct measures of muscle quality, muscle strength, physical performance, or sarcopenia. Quantitative pooling was not performed because the available studies differed in population, intervention, comparator, imaging-derived outcome definition, anatomical assessment, measurement scale, and variance reporting. Abbreviations: MRI, magnetic resonance imaging; GLP-1, glucagon-like peptide-1.
